# Low prevalence of *Moraxella catarrhalis* in the patients who suffered from conjunctivitis in the southwest of Iran

**DOI:** 10.1186/s13104-020-05389-4

**Published:** 2020-11-25

**Authors:** Ahmad Farajzadeh Sheikh, Mustafa Feghhi, Maryam Torabipour, Morteza Saki, Hojat Veisi

**Affiliations:** 1 Department of Microbiology, Faculty of Medicine, Ahvaz Jundishapur University of Medical Sciences, Ahvaz, Iran; 2 Department of Ophthalmology, Emam Khomeini Hospital, Ahvaz Jundishapur University of Medical Sciences, Ahvaz, Iran; 3 Student Research Committee, Ahvaz Jundishapur University of Medical Sciences, Ahvaz, Iran

**Keywords:** *Moraxella catarrhalis*, Conjunctivitis, Ocular infection, Iran

## Abstract

**Objective:**

*Moraxella catarrhalis* is a non-motile Gram-negative diplococcus bacterium that contributed to several human infections including conjunctivitis. This study aimed to reveal the prevalence of *M. catarrhali*s in patients who suffered from conjunctivitis in Ahvaz city, southwest of Iran.

**Results:**

Out of 100 conjunctiva swab specimens, *M. catarrhalis* was isolated only from one (1%) conjunctivitis cases using the culture method. This strain was isolated from a 34 years old female patient. Also, the results of the polymerase chain reaction (PCR) were in agreement with the culture method, and the specimen that showed positive culture was also positive for specific gene of *M. catarrhalis*. The remaining 99 specimens did not show positive results with any of the culture and PCR methods.

## Introduction

*Moraxella catarrhalis* is a Gram-negative, diplococcus bacterium that accounts for many pathologies of humans including otitis media, sinusitis, pneumonia, and conjunctivitis [1–3]. The inflammation of the membrane of eyelids is called conjunctivitis [4]. Several numbers of infectious pathogens including bacteria (*Staphylococcus aureus*, *Haemophilus influenzae*, *Streptococcus pneumoniae*, and *Moraxella catarrhalis*) and viruses (adenoviruses, herpes simplex virus, varicella-zoster virus, and *Molluscum contagiosum*) are contributed to the majority of conjunctivitis cases [5]. The incidence of viral conjunctivitis is higher than bacterial conjunctivitis in adults, whereas bacterial conjunctivitis is more common in children [6].

In total, bacterial conjunctivitis is accountable for 50–75% of cases in children [6]. *Staphylococcus* spp, *S. pneumoniae*, and *H. influenzae* account for the highest causes of bacterial conjunctivitis [5, 6]. In previous studies, the non-typeable *H. influenzae* (NTHi) had a frequency rate of 61.8% in children with infective conjunctivitis, followed by *S. pneumoniae* (28.2%), and *M. catarrhalis* (19.1%) [7]. In the last century, *M. catarrhalis* has been considered an emerging human pathogen [8]. This bacterium is resistant to penicillin due to its BRO-1 and BRO-2 beta-lactamases [8]. Conjunctivitis caused by *M. catarrhalis* is generally non-exudative, persistent, and with no sign of redness [9]. Currently, medical system laboratories do not have an identical sensitive method for differentiating *Moraxella* species to genus and species levels in eye infections [3]. Although the prevalence of *Moraxella* species in ocular infections has been rarely reported (0.8–19.1%) [3, 10], since there was no epidemiological information in this field in the geographical region of southwestern Iran, this study aimed to investigate the phenotypic and molecular presence of *M. catarrhalis* in conjunctivitis samples collected from patients admitted to one of the main referral ocular infections centers of southwestern Iran.

## Main text

### Methods

#### Sample collection and phenotypic detection of bacteria

In this study, which was performed from May 2013 to August 2016, the conjunctival samples were collected by sterile cotton-wool swabs (2 swabs) from each patient who suffered from infective conjunctivitis and admitted as outpatients to the Ophthalmology Division of Emam Khomeini Hospital in Ahvaz, southwestern Iran. The cases were selected and confirmed by the ophthalmologist attending physician. All cases with a history of antibiotics use 15 days before the study were excluded from research. The swab specimens were directly inoculated on blood agar (Merck, Darmstadt, Germany) with 5% defibrinated sheep blood (Bahar Afshan, Tehran, Iran) and incubated at 37 °C with 5% CO_2_ for 24–48 h. After overnight incubation, positive growth and suspected colonies were further investigated by colony morphology (smooth, white, opaque), Gram staining, oxidase, catalase, DNase, reduction of nitrate to nitrite, and carbohydrate fermentation tests such as sucrose, glucose, and lactose [11]. The suspected colonies were stocked in trypticase soy broth (Merck, Germany) containing 20% (v/v) glycerol and frozen at − 80 °C. The isolates were finally confirmed by polymerase chain reaction (PCR) assay.

## Molecular investigation

### DNA extraction and PCR assay

The DNA extraction of bacterial isolates and conjunctival swabs was done by the boiling method and QIAamp DNA Mini Kit (QIAGEN GmbH, Hilden, Germany), respectively [12–14]. The PCR was done to amplify a 140 bp sequence of *M. catarrhalis* using MCAT1 (5′-TTGGCTTGTGCT AAAATATC-3′) and MCAT2 (5′-GTCATCGCTATCATTCACCT-3′) primers as previously described by Post et al. [15]. The PCR reaction was carried out in a final volume of 25 µl using a thermal gradient cycler (Eppendorf Co., Germany) with the following procedure: 2.5 µl of 10× buffer (10 mM Tris-HCl, 50 mM KCl), 1.5 mM MgCl_2_, 200 µM of each dNTPs, 0.4 µM of each forward and reverse primers, 1 U of Taq polymerase, 3 µl of the DNA template, and sterilized distilled water to complete the reaction volume. The PCR program was optimized as initial denaturation at 95 °C for 5 min, 34 cycles of denaturation at 95 °C for 50s, annealing at 55 °C for 50s, extension at 72 °C for 50s, and the final extension time at 72 °C for 5 min. The PCR amplicons were visualized by electrophoresis (80 V/60 min) on 1.5% agarose gel in 1× Tris Borate-EDTA buffer containing ethidium bromide (0.5 µg/ml). *M. catarrhalis* ATCC 25238 and distilled water were used as positive and negative controls respectively.

## Results

**Fig. 1:**
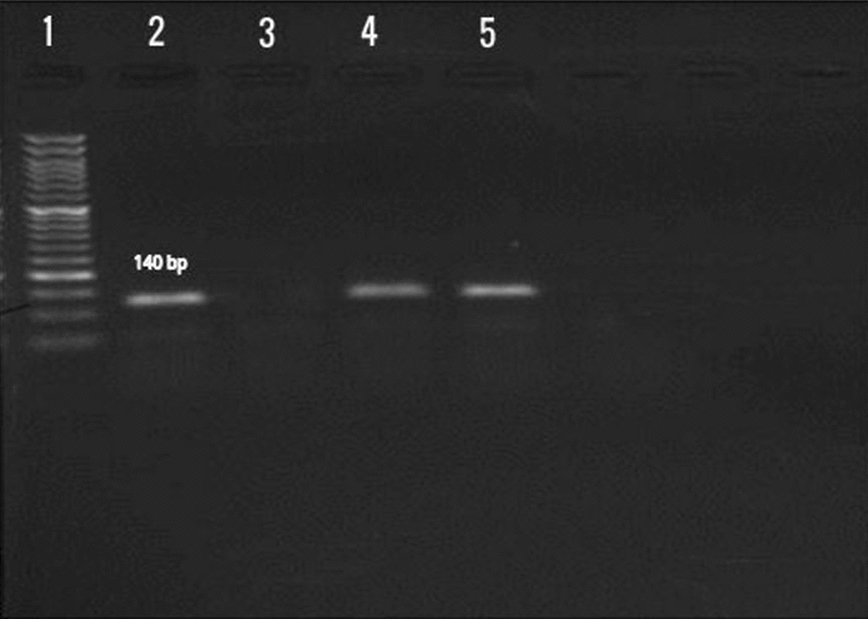
Lanes 1: ladder of 50 bp; lane 2: positive control, lane 3: negative control, lane 4: the amplicon of bacterial isolate, lane 5: the amplicon of conjunctival sample

In this study, a total of 100 patients were enrolled from which 65 and 35 were males and females, respectively. The mean age was 45 years (range 7–76 years). The result of the culture of conjunctival swab specimens showed that only one sample was positive for *M. catarrhalis* growth. This isolate had a positive reaction for catalase, oxidase, nitrate reduction, and DNase (Table 1); also exhibited a positive band of 140 bp in PCR assay (Fig. 1). *M. catarrhalis* was isolated from a 34 year old female patient. The other 99 specimens were negative for *M. catarrhalis* by culture method. Also, the PCR method detected the *M. catarrhalis* in only one of the extracted DNA samples that belong to the culture positive specimen. The remaining 99 specimens were negative for the specific band of *M. catarrhalis* by PCR.

**Table 1.** Biochemical characteristics of *Moraxella catarrhalis* isolated from conjunctival swab.

Tests	Results
Catalase test	+
Oxidase test	+
Citrate test	−
Indole test	−
Glucose	−
Lactose	−
Sucrose	−
Mannitol	−
Reduction of nitrate	+
DNAse production	+

## Discussion

Conjunctivitis is one of the main causes of redness and discharge of the eyes, and its prevalence varies according to the patients’ gender and age, and different seasons of the year [16]. To the best of our knowledge, there is little evidence available in Iran about conjunctivitis caused by *M. catarrhalis*, although some studies have been performed on the bacterial and viral agents that cause the disease [17, 18]. We observed a higher occurrence of conjunctivitis in male (65%) when compared to female (35%) patients. A similar result was observed in an Italian independent study by Petrillo et al. [19]. In this study, the results of culture and PCR methods revealed the occurrence rate of 1% for *M. catarrhalis* in conjunctivitis samples. In a study by Afjeiee et al. [17] from Tehran, Iran, of the 241 neonates with clinical signs of conjunctivitis, the most frequent isolates were coagulase-negative *Staphylococci* (n = 130, 53.9%), followed by *Chlamydia trachomatis* (n =  40, 16.6%). They did not detect *M. catarrhalis* by routine culture method. Another report by Bhattacharyya et al. [20] from India revealed a frequency rate of 4.5% for *M. catarrhalis* among 110 culture- positive specimens of acute bacterial conjunctivitis that was higher than the current study. Also, Iwalokun et al. [21] from Nigeria reported an occurrence rate of 4.5% for *Moraxella* spp, among 83 conjunctival specimens.

So far, *M. catarrhalis* has been reported from several eye infection types, such as blepharitis, conjunctivitis, dacryocystitis, keratitis, and endophthalmitis [10]. The systematic review by Teweldemedhin et al. [10] documented a higher incidence of *Moraxella* spp., particularly *M. catarrhalis* in keratitis when compared to other eye infections. In a study by Jyoti et al. [22] from India, the prevalence of *M. catarrhalis* was reported as 0.4% in conjunctivitis specimens that was lower than this study. In previous reports from various countries of the world, the coagulase-negative *Staphylococci* and *S. aureus* were among the most prevalent species isolated from eye infections, including conjunctivitis [17, 19, 22].

Another point to consider about the bacterium *M. catarrhalis* is that its prevalence has rarely been evaluated by precise and sensitive molecular methods including PCR, real-time PCR, and loop-mediated isothermal amplification in ocular infections; while most studies of the presence of this bacterium in the respiratory specimens were done with molecular assessment in parallel with the conventional culture method [3, 23]. This may indicate that the actual prevalence of this bacterium in ocular infections is lower than the original rate, as the use of molecular methods is much more specific and sensitive than the conventional culture method. Some previous studies that used conventional culture method, matrix assisted laser desorption/ionization time-of-flight mass spectrometry (MALDI-TOF MS), VITEK 2 system, and DNA sequencing have been reported several *Moraxella* species including *M. nonliquefaciens*, *M. lacunata*, *M. osloensis*, *M. atlantae*, and *M. catarrhalis* as a cause of ocular infections [3, 24, 25].

## Conclusion

Although the *M. catarrhalis* was identified with a low rate as a causative agent of conjunctivitis in our region, this study can be considered as a starting point for investigation of this pathogen in all types of eye infections with larger sample size and with a multicentral design.

## Limitation

In this study, the prevalence and incidence rates of other bacterial or viral pathogens that may have been responsible for conjunctivitis were not investigated due to the very low financial sources that can be considered as a limitation of the current research.

## Data Availability

The data of the current study are available from the corresponding authors on reasonable request.

## Abbreviations

MALDI-TOF MS: Matrix assisted laser desorption/ionization time-of-flight mass spectrometry
NTHi: Nontypeable *Haemophilus influenza*
PCR: Polymerase chain reaction
